# The genetic variations in DNA repair genes *ERCC2* and *XRCC1* were associated with the overall survival of advanced non‐small‐cell lung cancer patients

**DOI:** 10.1002/cam4.822

**Published:** 2016-07-27

**Authors:** Suhan Wang, Jianzhong Wang, Yansen Bai, Qing Wang, Li Liu, Kai Zhang, Xiaohua Hong, Qifei Deng, Xiaomin Zhang, Meian He, Tangchun Wu, Ping Xu, Huan Guo

**Affiliations:** ^1^ Department of Occupational and Environmental Health and Ministry of Education Key Lab for Environment and HealthSchool of Public HealthTongji Medical College, Huazhong University of Science and TechnologyWuhan430030China; ^2^ Department of OncologyWuhan Iron and Steel (Group) Corporation Staff‐Worker HospitalWuhan430085China; ^3^ Department of OncologyCancer CenterUnion Hospital, Huazhong University of Science and TechnologyWuhan430022China

**Keywords:** DNA repair, genetic variation, lung cancer, prognosis

## Abstract

It was reported that DNA repair can confer cancer cell resistance to therapeutic treatments by activating antiapoptotic cellular defense. We hypothesized that genetic variants of DNA repair genes may be associated with lung cancer prognosis. Seventeen tagging single‐nucleotide polymorphism (tagSNPs) selected from 12 DNA repair genes were genotyped in 280 advanced non‐small‐cell lung cancer (NSCLC) patients by TaqMan assay. The associations of these SNPs and overall survival of advanced NSCLC patients were investigated. Advanced NSCLC patients carrying *ERCC2* rs50872 CT+TT genotypes had significantly longer median survival time (MST) and decreased death risk than patients with rs50872 CC genotype [log‐rank *P *=* *0.031; adjusted HR(95% CI) = 0.73 (0.55–0.98), *P *=* *0.033]. These effects were mainly seen among younger patients (≤65 years old) [HR(95% CI) = 0.57 (0.37–0.87), *P *=* *0.010], patients without surgery [HR(95% CI) = 0.68 (0.47–0.98), *P *=* *0.036] but with chemotherapy [HR(95% CI) = 0.64 (0.46–0.91), *P *=* *0.012] or radiotherapy [HR(95% CI) = 0.58 (0.38–0.89), *P *=* *0.013]. Meanwhile, compared to advanced NSCLC patients with rs25487 GG genotype, patients carrying *XRCC1* rs25487 GA+AA genotypes had significantly shorter MST (MST = 11.7 vs. 16.7, log‐rank *P *=* *0.048). In addition, advanced NSCLC patients carrying the *ERCC2* rs50872 CC in combination with *XRCC1* rs25487 GA+AA genotype had the shortest MST (11.2 month) and highest death risk [HR(95% CI) = 1.70 (1.15–2.52), *P *=* *0.008] when compared with those carrying rs50872 CT+TT and rs25487 GG genotype (MST = 22.0 month). The *ERCC2* rs50872 T allele was associated with favorable but *XRCC1* rs25487 A allele with bad survival for advanced NSCLC in Chinese population, which may offer novel biomarkers for predicting clinical outcomes.

## Introduction

Lung cancer is the leading cause of cancer‐related mortality in China and worldwide 1. Non‐small‐cell lung cancer (NSCLC) is the most common subtype that accounts for 85% of all lung cancer patients. The majority of NSCLC patients were diagnosed at an advanced tumor stage and lost the opportunity of surgical resection 2. For more than two decades, platinum‐based combination treatment is a standard treatment for advanced NSCLC. However, the effectiveness has apparently reached a plateau, and the overall survival rate has still been extremely poor. Populations with diverse genetic variations in candidate pathways have been proposed to affect the susceptibility to cancer development, response efficiency to cancer treatment, and survival outcomes for lung cancer patients 3.

DNA repair capacity (DRC) is a double‐edged sword in the etiology and response to clinical therapies for various cancers. The individual's susceptibility to cancer risk can be drastically increased due to defect in DNA repair system 4, 5. On the other hand, increased DRC may influence the sensitivity of tumor cells to chemo‐ and radiotherapy and thus affect therapeutic efficacy by permitting cancer cells to fix DNA damages aroused by these agents 5, 6. Nucleotide excision repair (NER) and base excision repair (BER) are two major DNA repair pathways those involve coordination of numerous genes. Single‐nucleotide polymorphisms (SNPs) in DNA repair genes may modulate DNA repair capacity via influencing protein expression or activities, and therefore affecting the therapeutic response for lung cancer patients 7, 8, 9, 10. As a result, identifying special genetic biomarkers in DNA repair pathways to guide personalized therapy strategy may minimize therapy resistance and improve the clinical outcome of NSCLC patients.

Thus, 12 DNA repair genes were chosen for analysis in this study, including nine key processor genes (*RRM1*,* ERCC1*,* ERCC2*,* XPA*,* XPB*,* XPF*,* XPG*,* CSB*, and *DDB2*) in the NER pathway, and three key processor genes (*XRCC1*,* FEN1*, and *APEX1*) from the BER pathway. A total of 17 SNPs (general information shown in Table 1) were selected from the above genes and their associations with the overall survival of advanced NSCLC patients were further investigated.

**Table 1:** Selected DNA repair genes and single‐nucleotide polymorphisms in this study

Pathway	Gene	SNP	Chr	Chr position^a^	Alleles	Function	MAF
Nucleotide excision repair							
*RRM1*		rs11030918	11	4094257	T/C	5′near gene	0.302
		rs12806698	11	4094744	C/A	5′UTR	0.227
*ERCC1*		rs11615	19	45420395	C/T	Asn118Asn	0.331
		rs3212986	19	45409478	G/T	3′UTR	0.295
*ERCC2*		rs13181	19	45351661	T/G	Lys751Gln	0.237
		rs50872	19	45359191	C/T	Intron	0.182
*XPA*		rs1800975	9	97697296	G/A	Intron	0.354
*XPB*		rs2276583	2	127257108	A/G	3′near gene	0.379
*XPF*		rs1799797	16	13920136	T/A	5′near gene	0.220
*XPG*		rs17655	13	102875652	G/C	Asp1104His	0.361
*CSB*		rs3793784	10	49539493	C/G	5′near gene	0.238
*DDB2*		rs2029298	11	47213167	C/T	5′near gene	0.445
		rs3781619	11	47233766	A/G	Intron	0.357
Base excision repair							
*XRCC1*		rs25487	19	43551574	G/A	Arg399Gln	0.260
*FEN1*		rs174538	11	61792609	G/A	5′near gene	0.282
		rs4246215	11	61796827	G/T	3′UTR	0.303
*APEX1*		rs1130409	14	20456995	G/T	Asp148Glu	0.376

a dbSNP Chromosome Report, GRCh38.

## Materials and Methods

### Ethics statement

The study subjects provided their written informed consent after a clear explanation of study objective. All subjects were genetically unrelated ethnic Han Chinese and this study was approved by the Institutional Review Board of Tongji Medical College, Huazhong University of Science and Technology.

### Study population

We recruited 405 lung cancer cases from Wuhan Steel Group/Corporation Staff‐Worker Hospital between January 2003 and December 2010 in Wuhan City, Hubei Province of Central China. After being diagnosed with lung cancer, the patients received treatment at the same hospital until they died from the disease, and more than 98% patients kept good follow‐up. The 280 advanced NSCLC patients who had completed follow‐up and clinical information were included in the survival analysis. The TNM stage classification was evaluated according to the Staging Manual of AJCC/UICC. We followed up the patients through telephone calls every 3 months until 31 December 2010, and acquired date of death from the hospital records and patients’ families via the follow‐up telephone calls. We considered patients who were still alive on 31 December 2010 as censored, and calculated the survival time for each patient from the date when patients were confirmed diagnosed of lung cancer until the date of death or the last follow‐up. The large part of the study patients have been published in our previous study 11.

Written informed consent for storage and use of blood samples, and for obtaining medical records information during follow‐up were provided by all patients. Information on demographic characteristics, tobacco smoking, alcohol consumption, medical history, and family history of cancer were collected through an interview using a pretested questionnaire. Individuals who had smoked <1 cigarette per day for less than 1 year in their entire lifetime were defined as nonsmokers; those who had stopped smoking for more than 1 year were considered as former smokers; otherwise, those who were still smoking in the previous year were defined as current smokers.

### DNA extraction and genotyping

Genomic DNA was extracted using the Gentra puregene blood kit (Qiagen, Hilden, Germany) following the manufacturer's instructions. In this study, genotyping of SNPs in all subjects were carried out by the TaqMan method using the ABI 7900HT Sequence Detection System (Applied Biosystems). All primers and probes were ordered from Applied Biosystems. The sequences of primers and probes are available in Supplementary Table 1. The cycling conditions were conducted as described in detail previously 11: 50°C for 2 min, 95°C for 10 min, and followed by 45 cycles of 95°C for 15 sec and 60°C for 1 min. For quality control, we randomly selected 5% samples as repeated trials, and the repeated results were identical as the former results..

### Statistical analysis

The Kaplan–Meier method and log‐rank test were used to calculate and compare the median survival time (MST) for patients with different age, gender, smoking status, histology, TNM stage, therapy treatments of surgical resection, chemotherapy, radiotherapy, and different genotypes. The associations between SNPs and death risk of advanced NSCLC patients were estimated using the multivariate Cox regression models, with adjustment of age, smoking status, histology, TNM stage, and therapy treatments of surgical resection, chemotherapy, and radiotherapy. The effect modifications by patient characteristics and clinical features (age, smoking status, histology, TNM stage, and therapy treatment of surgical resection, chemotherapy, and radiotherapy) on the effects of SNPs on death risk of advanced NSCLC patients were assessed using the Wald test in the multivariate Cox proportional hazards regression models after adjusting for the confounders. All analyses were conducted on the SPSS 20.0 software (SPSS Inc., Chicago, IL) and a two‐side *P *<* *0.05 was considered statistically significant.

## Results

### Patient characteristics

The demographic and clinical characteristics of the 280 advanced NSCLC patients who had completed the follow‐up information are listed in Table 2. For these patients, the mean age was 64.28 ± 9.30 years, 214 (76.4%) patients died of lung cancer, 83 (29.6%) received surgical operations, 215 (76.8%) received chemotherapies, and 131 (46.8%) received radiotherapies. The Kaplan–Meier analysis, log‐rank test, and univariate Cox analysis showed that elder patients aged >65 (MST = 12.2 vs. 17.9, log‐rank *P *=* *0.001) and patients with an advanced stage (MST = 10.8 vs. 16.7 vs. 19.1, log‐rank *P *<* *0.001) had a significantly shorter MST and an increased risk of death. Similarly, patients who received surgical operation (MST = 16.9 vs. 12.7, Log‐rank *P *=* *0.019) and chemotherapy (MST = 16.0 vs. 11.0, Log‐rank *P *=* *0.035) had more clinical benefit than patients who did not receive surgical operation or chemotherapy, respectively. However, no significant effects were found for gender, smoking status, histological subtype, and radiotherapy on MST and death risk for advanced NSCLC patients.

**Table 2:** Patient characteristics and clinical features

Variables	Lung cancer patients, *n*(%)	Deaths	MST (month)	Log‐rank *P*	HR (95% CI) a
	(*N* = 280)	(*n* = 214)			
Age					
≤65	136 (48.6)	98	17.9	0.001	1.00 (Reference)
>65	144 (51.4)	116	12.2		1.58 (1.20–2.07)
Sex					
Male	241 (86.1)	183	13.3	0.089	1.00 (Reference)
Female	39 (13.9)	31	25.2		0.72 (0.49–1.06)
Smoking					
Never	44 (15.7)	33	20.7	0.200	1.00 (Reference)
Former smokers	150 (53.6)	115	13.7		1.42 (0.96–2.10)
Current smokers	86 (30.7)	66	13.4		1.27 (0.84–1.94)
Histology					
Adenocarcinoma	114 (40.7)	85	17.4	0.054	1.00 (Reference)
SCC	78 (27.9)	58	16.5		0.90 (0.64–1.25)
Others^b^	88 (31.4)	71	10.6		1.40 (0.98–1.84)
Stage					
IIIA	70 (25.0)	47	19.1	<0.001	1.00 (Reference)
IIIB	77 (27.5)	55	16.7		1.27 (0.86–1.87)
IV	133 (47.5)	112	10.8		1.92 (1.36–2.70)
Surgery					
No	197 (70.4)	149	12.7	0.019	1.00 (Reference)
Yes	83 (29.6)	65	16.9		0.71 (0.53–0.95)
Chemotherapy					
No	65 (23.2)	49	11.0	0.035	1.00 (Reference)
Yes	215 (76.8)	165	16.0		0.71 (0.52–0.98)
Radiotherapy					
No	149 (53.2)	110	13.3	0.368	1.00 (Reference)
Yes	131 (46.8)	104	15.3		0.88 (0.68–1.16)

HR, hazard ratio.

a Data were calculated by univariate Cox regression analysis.

b Others include large cell, bronchoalveolar, mixed cell, undifferentiated and pathologic, not otherwise specified carcinomas.

### Associations of SNPs and survival of advanced NSCLC patients

**Figure 1:**
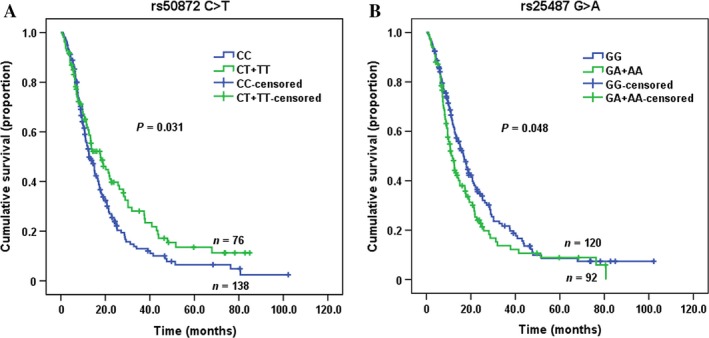
Kaplan–Meier survival curves for advanced NSCLC patients by *ERCC2* rs50872 (A) and *XRCC1* rs25487 (B) genotypes. NSCLC, non‐small‐cell lung cancer.

As shown in Table 3, the Kaplan–Meier method and log‐rank test showed that the advanced patients carrying the *ERCC2* rs50872 CT and CT+TT genotypes had the MST of 18.0 and 17.8 months, respectively, which were significantly longer than the survival time of rs50872 CC genotype carriers (MST = 12.7, log‐rank *P *=* *0.034 and 0.031, respectively) (Fig. 1). The multivariate Cox regression models revealed that the adjusted hazard ratio (HR) and 95% CI of death risk was 0.72 (0.54–0.97) for rs50872 CT, 0.82 (0.37–1.81) for rs50872 TT, and 0.73 (0.55–0.98) for rs50872 CT+TT genotype, compared with the rs50872 CC genotype (Table 3). There was a dose–response effect of the rs50872 T allele in reducing death risk (*P*_trend_  = 0.018). Meanwhile, the Kaplan–Meier method and log‐rank test showed that the advanced patients carrying the *XRCC1* rs25487 GA and GA+AA genotypes had the MST of 11.2 and 11.7 months, respectively, which were significantly shorter than the survival time of rs25487 GG genotype carriers (MST = 16.7, log‐rank *P *=* *0.038 and 0.048, respectively) (Table 3, Fig. 1). Patients carrying *XRCC1* rs25487 GA+AA genotype had a marginally increased risk of death than those with rs25487 GG genotype [HR (95% CI) = 1.29 (0.97–1.70)] (Table 3). For all other polymorphisms, we did not find any association of their genotypes with the survival outcomes of advanced NSCLC patients.

**Table 3:** Associations between SNP genotypes and survival of patients with advanced non‐small‐cell lung cancer

Genes	SNPs	HR (95% CI)^a^	*P* a	Lung cancerpatients (*N* = 280)	Deaths(*n* = 214)	MST(month)	Log‐rank *P*
NER pathway genes							
*RRM1*	rs11030918						
	TT	1.00 (Reference)		126 (45.0)	98	16.7	
	TC	0.99 (0.74–1.32)	0.928	123 (43.9)	93	13.4	0.790
	CC	1.06 (0.67–1.70)	0.796	30 (10.7)	23	11.7	0.929
	rs12806698						
	CC	1.00 (Reference)		133 (47.5)	105	15.8	
	CA+AA	1.00 (0.78–1.32)	0.993	146 (52.2)	109	12.6	0.916
*ERCC1*	rs11615						
	CC	1.00 (Reference)		166 (59.3)	127	13.9	
	CT+TT	0.89 (0.67–1.18)	0.398	113 (40.3)	87	13.7	0.895
	rs3212986						
	GG	1.00 (Reference)		103 (36.8)	80	13.7	
	GT	1.19 (0.87–1.62)	0.283	134 (47.9)	100	15.3	0.734
	TT	0.97 (0.65–1.46)	0.885	42 (15.0)	34	12	0.494
*ERCC2*	rs13181						
	TT	1.00 (Reference)		236 (84.3)	179	13.4	
	TG+GG	0.89 (0.62–1.28)	0.517	43 (15.4)	35	18.4	0.457
	rs50872						
	CC	1.00 (Reference)		171 (61.1)	138	12.7	
	CT	0.72 (0.54–0.97)	0.032	95 (33.9)	69	18.0	0.034
	TT	0.82 (0.37–1.81)	0.625	13 (4.6)	7	13.7	0.490
	CT+TT	0.73 (0.55–0.98)	0.033	108 (38.5)	76	17.8	0.031
		*P*_trend_	0.018				
*XPA*	rs1800975						
	GG	1.00 (Reference)		80 (28.6)	64	14.5	
	GA	0.95 (0.69–1.31)	0.763	133 (47.5)	106	13.4	0.978
	AA	0.87 (0.59–1.28)	0.476	66 (23.6)	44	17.8	0.427
*XPB*	rs2276583						
	AA	1.00 (Reference)		97 (34.6)	73	16.5	
	AG	1.03 (0.76–1.39)	0.870	151 (53.9)	118	12.7	0.183
	GG	0.89 (0.55–1.44)	0.647	31 (11.1)	23	21	0.470
*XPF*	rs1799797						
	TT	1.00 (Reference)		168 (60)	130	14.5	
	TA+AA	1.20 (0.91–1.60)	0.203	111 (39.6)	84	13.7	0.823
*XPG*	rs17655						
	GG	1.00 (Reference)		78 (27.9)	58	16.5	
	GC	1.17 (0.84–1.63)	0.357	144 (51.4)	113	13.3	0.113
	CC	1.03 (0.69–1.53)	0.905	57 (20.4)	43	14.5	0.550
*CSB*	rs3793784						
	CC	1.00 (Reference)		135 (48.2)	105	12.7	
	CG	0.98 (0.73–1.32)	0.915	116 (41.4)	87	14.5	0.654
	GG	1.24 (0.77–2.00)	0.375	28 (10.0)	22	15	0.945
*DDB2*	rs3781619						
	AA	1.00 (Reference)		94 (33.6)	74	14.7	
	AG	0.93 (0.69–1.27)	0.659	140 (50.0)	104	13.3	0.985
	GG	0.88 (0.59–1.32)	0.545	45 (16.1)	36	15	0.813
	rs2029298						
	CC	1.00 (Reference)		129 (46.1)	102	13.4	
	CT	0.91 (0.68–1.22)	0.511	118 (42.1)	87	16.7	0.343
	TT	1.03 (0.65–1.62)	0.910	32 (11.4)	25	13.3	0.911
BER pathway genes							
*XRCC1*	rs25487						
	GG	1.00 (Reference)		159 (56.8)	120	16.7	
	GA	1.29 (0.95–1.74)	0.099	95 (33.9)	75	11.2	0.038
	AA	1.29 (0.77–2.16)	0.341	22 (7.9)	17	12.5	0.593
	GA+AA	1.29 (0.97–1.70)	0.075	117 (41.8)	92	11.7	0.048
*FEN1*	rs174538						
	GG	1.00 (Reference)		108 (38.6)	86	14.2	
	GA	1.12 (0.81–1.53)	0.500	121 (43.2)	92	13.4	0.698
	AA	0.83 (0.55–1.24)	0.368	50 (17.9)	36	16.7	0.435
	rs4246215						
	GG	1.00 (Reference)		106 (37.9)	86	14.2	
	GT	1.11 (0.81–1.52)	0.533	119 (42.5)	91	13.4	0.713
	TT	0.81 (0.55–1.21)	0.311	54 (19.3)	37	17.9	0.390
*APEX1*	rs1130409						
	GG	1.00 (Reference)		86 (30.7)	64	13.7	
	GT	0.91 (0.66–1.25)	0.553	144 (51.4)	108	17.0	0.681
	TT	1.31 (0.87–1.99)	0.199	47 (16.8)	40	10.0	0.267

HR, hazard ratio; NER, Nucleotide excision repair; BER, base excision repair.

a The Cox regression analysis was adjusted for age, sex, smoking status, histology, TNM stage, surgery, chemotherapy, and radiotherapy status. Note: Survival analyses were determined for haplotypes or diplotypes >10% frequency.

### Stratification analyses for ERCC2 rs50872 and XRCC1 rs25487 on survival of advanced NSCLC patients

The advanced NSCLC patients were further stratified by their features of age, smoking status, histology, TNM stage, and therapy treatments. The protective effects of rs50872T allele were more obvious in subjects aged ≤65 years old [HR (95% CI) = 0.57 (0.37–0.87), *P *=* *0.010], patients without surgery [HR (95% CI) = 0.68 (0.47–0.98), *P *=* *0.036], but who underwent chemotherapy[HR (95% CI) = 0.64 (0.46–0.91), *P *=* *0.012] and radiotherapy [HR (95% CI) = 0.58 (0.38–0.89), *P *=* *0.013] (Table 4).

**Table 4:** Stratification analyses for associations between SNP genotypes and overall survival of advanced non‐small‐cell lung cancer patients

Variables	*ERCC2*‐rs50872CC		*ERCC2‐*rs50872‐CT+TT					*P*_interaction_ a	*XRCC1*‐rs25487GG		*XRCC1*‐rs25487‐GA+AA					*P*_interaction_ a
	n_p_/n_d_	MST	n_p_/n_d_	MST	Log‐rank *P*	HR(95% CI)^a^	*P* a		n_p_/n_d_	MST	n_p_/n_d_	MST	Log‐rank *P*	HR(95% CI)^a^	*P* a	
Age								0.314								0.773
≤65	81/62	16.7	54/36	28.1	0.077	0.57 (0.37–0.87)	0.010		77/54	20.7	57/44	15.0	0.121	1.15 (0.76–1.73)	0.516	
>65	90/76	11.3	54/40	13.3	0.377	0.82 (0.55–1.23)	0.332		82/66	13.5	60/48	9.5	0.193	1.36 (0.93–1.99)	0.117	
Sex								0.816								0.561
Male	146/116	12.5	94/67	13.7	0.104	0.73 (0.54–1.00)	0.050		132/98	15.8	105/83	11.1	0.068	1.34 (1.00–1.81)	0.052	
Female	25/22	23.9	9/14	28.3	0.106	0.47 (0.18–1.20)	0.115		27/22	25.2	12/9	23.9	0.685	1.10 (0.48–2.49)	0.824	
Smoking								0.770								0.663
Never‐smokers	24/21	12.7	147/117	28.9	0.179	0.50 (0.23–1.10)	0.083		27/20	28.9	17/13	8.7	0.157	1.35 (0.57–3.20)	0.499	
Smokers	12/20	12.9	88/64	13.9	0.088	0.73 (0.53–1.00)	0.053		132/100	15.8	100/79	12.0	0.175	1.27 (0.94–1.72)	0.117	
Histology								0.154								0.352
Adenocarcinoma	74/58	15.0	40/27	21.3	0.088	0.77 (0.47–1.24)	0.279		64/48	18.4	50/37	15.0	0.728	1.02 (0.65–1.59)	0.947	
SCC	47/35	16.5	31/23	13.7	0.713	1.18 (0.68–2.02)	0.562		48/35	17.9	30/23	12.5	0.046	1.68 (0.91–3.09)	0.095	
Others^b^	50/45	9.6	37/26	13.3	0.018	0.50 (0.27–0.91)	0.023		47/37	11.3	37/32	9.0	0.349	1.52 (0.92–2.53)	0.103	
Stage								0.467								0.136
IIIA	44/31	19.0	26/16	25.7	0.323	0.67 (0.33–1.34)	0.252		41/24	22.8	29/23	15.0	0.078	1.56 (0.78–3.14)	0.210	
IIIB	51/38	14.5	25/17	28.1	0.059	0.65 (0.35–1.20)	0.170		43/32	18.4	32/22	13.4	0.125	1.60 (0.88–2.94)	0.125	
IV	76/69	10.6	57/43	12.6	0.240	0.76 (0.51–1.14)	0.187		75/64	11.7	56/47	10.0	0.798	1.14 (0.76–1.71)	0.535	
Surgery								0.543								0.973
No	117/95	12.7	79/54	13.4	0.088	0.68 (0.47–0.98)	0.036		110/83	15.8	83/64	10.5	0.057	1.40 (0.99–1.97)	0.058	
Yes	54/43	15.0	29/22	25.7	0.190	0.82 (0.46–1.47)	0.505		49/37	18.0	34/28	13.9	0.378	1.29 (0.76–2.19)	0.343	
Chemotherapy								0.764								0.986
No	31/24	11.0	33/25	10.7	0.663	0.92 (0.49–1.71)	0.793		31/23	12.2	30/24	8.3	0.265	1.62 (0.81–3.27)	0.176	
Yes	140/114	15.0	75/51	21.7	0.011	0.64 (0.46–0.91)	0.012		128/97	17.9	87/68	12.7	0.117	1.36 (0.99–1.87)	0.059	
Radiotherapy								0.259								0.053
No	86/66	14.7	62/44	11.5	0.659	0.88 (0.58–1.34)	0.555		83/59	17.9	62/49	10.8	0.008	1.77 (1.19–2.63)	0.005	
Yes	85/72	12.0	46/32	26.3	0.008	0.58 (0.38–0.89)	0.013		76/61	16.0	55/43	12.5	0.796	0.97 (0.65–1.45)	0.888	

a The Cox regression analysis was adjusted for age, sex, smoking status, histology, TNM stage, surgery, chemotherapy, and radiotherapy status when appropriate.

b Others include large cell, bronchoalveolar, mixed cell, undifferentiated and pathologic, not otherwise specified carcinomas.

For the *XRCC1* rs25487 polymorphism, the effect of rs25487 GA+AA genotype on elevated death risk of NSCLC patients was significant in the patients without radiotherapy [HR (95% CI) = 1.77 (1.19–2.63), *P *=* *0.005]. In addition, the radiotherapy can marginally modify the effect of *XRCC1* rs25487 GA+AA genotype on death risk for advanced NSCLC patients (*P*_interaction_ = 0.053). We also observed the marginal association between rs25487 GA+AA genotype with the increased death risk for males [HR (95% CI) = 1.34 (1.00–1.81), *P *=* *0.052], advanced NSCLC patients without surgery [HR (95% CI) = 1.40 (0.99–1.97), *P *=* *0.058] but those who underwent chemotherapy [HR (95% CI) = 1.36 (0.99–1.87), *P *=* *0.059] (Table 4).

### The combinative effects of ERCC2 rs50872 and XRCC1 rs25487 on survival of advanced NSCLC patients

We stratified the study patients by both *ERCC2* rs50872 and *XRCC1* rs25487 variants. Among patients with *ERCC2* rs50872CC genotype, those with *XRCC1* rs25487 GA+AA genotypes had increased death risk than those with rs25487GG genotype [HR (95% CI) = 1.67 (1.04–2.68), *P *=* *0.034]; but among patients with *ERCC2* rs50872CT+TT genotypes, the above effects of *XRCC1* rs25487 GA+AA on death risk for advanced NSCLC patients were eliminated [HR (95% CI) = 1.06 (0.75–1.50), *P *=* *0.741]. However, there was no significant interaction between *ERCC2* rs50872 and *XRCC1* rs25487 on death risk for advanced NSCLC patients (*P*_interaction_ = 0.134). We further analyzed the combinative effects of the two SNPs on the overall survival of advanced NSCLC patients. The Kaplan–Meier method and the Cox regression models showed that the advanced patients carrying the *ERCC2* rs50872 CC genotype in combination with *XRCC1* rs25487 GA+AA genotype had the shortest MST (11.2 month) and worst death risk [HR (95% CI) = 1.70 (1.15–2.52), *P *=* *0.008] when compared with those carrying rs50872 CT+TT and rs25487 GG genotype (MST = 22.0 month) (Table 5)**.**

**Table 5:** The combinative effects of *ERCC2* rs50872 and *XRCC1* rs25487 on the overall survival of advanced non‐small‐cell lung cancer patients

*ERCC2*rs50872	*XRCC1*rs25487	n_p_/n_d_	MST	Log‐rank *P*	HR(95% CI)^a^	*P* a	HR(95% CI)^a^	*P* a
CT+TT	GG	69/45	22.0	–	1.00 (Reference)	–	1.00 (Reference)	–
	GA+AA	37/30	12.6	0.008	1.67 (1.04–2.68)	0.034	1.67 (1.04–2.68)	0.034
CC	GG	90/75	15.3	0.004	1.00 (Reference)	–	1.60 (1.09–2.36)	0.017
	GA+AA	80/62	11.2	0.015	1.06 (0.75–1.50)	0.741	1.70 (1.15–2.52)	0.008

MST, median survival time; HR, hazard ratio.

a The Cox regression analysis was adjusted for age, sex, smoking status, histology, TNM stage, surgery, chemotherapy, and radiotherapy status.

## Discussion

This study investigated 13 SNPs in nine NER genes and four SNPs in three BER genes, and found that genetic variations of *ERCC2* and *XRCC1* may play important roles in predicting the overall survival of advanced NSCLC patients in Han Chinese. The *ERCC2* rs50872 T allele was associated with a favorable survival outcome for advanced NSCLC patients, and these effects were mainly seen in male patients, elder patients, and in patients without surgery but who underwent chemotherapy or radiotherapy. However, the *XRCC1* rs25487 A allele was associated with a bad survival outcome for advanced NSCLC patients, and these effects were mainly seen in male patients, and in patients who underwent chemotherapy but without surgery and radiotherapy. The advanced patients carrying the *ERCC2* rs50872 CC genotype in combination with *XRCC1* rs25487 GA+AA genotype had the shortest MST and highest death risk when compared with those carrying rs50872 CT+TT and rs25487 GG genotype. However, no significant associations were found for the other polymorphisms and survival outcomes of advanced NSCLC patients.

Exposure to environmental and endogenous carcinogens such as environmental chemical agents, ultraviolet light, and ionizing radiation can lead to a variety of DNA alterations 12. Most of these alterations may give rise to genetic instability, mutagenesis, and cell death if the alterations have not been repaired appropriately 12. DNA repair plays important roles in maintaining genome integrity, preventing carcinogenesis, and interindividual variability in platinum inactivation. The reduced DNA capacity is associated with increased response and improved survival to chemo‐ and radio‐ therapies that act by damaging DNA of cancer cells^13^. Given the possible effects on gene expression, we postulated that genetic polymorphisms of DNA repair genes might influence the individuals’ response to cancer therapies. Therefore, it is important to perform a pathway‐based analysis including DNA repair pathways that may affect the efficiency of response to cancer therapy.

NER is the major repair system for removing bulky DNA lesions such as monoadducts, cross‐links, and oxidative damages, especially those caused by cigarette smoking 13, 14, 15, 16. *ERCC2* is an integral member of the core transcription factor IIH via p44, and the ATP‐dependent DNA helicase activity of *ERCC2* opens the double helix in order to cut the damaged strand and remove the damaged DNA pieces.^13^, ^17^, ^18^, ^19^. One previous study in the Korean population suggested that the *ERCC2* rs50872 TT genotype was associated with a significantly poorer response and a poor prognostic factor in 129 NSCLC patients without surgery but treated with platinum‐based chemotherapy 13. This was the only study ever published about the *ERCC2* rs50872 polymorphism and lung cancer prognosis. However, on the contrary, our study suggested that the *ERCC2* rs50872 T allele was associated with a favorable prognosis for advanced NSCLC patients. These inconsistent results may be due to the patients’ heterogeneity and different social status between their study patients and ours.

A total of four SNPs in three BER genes were evaluated. The *XRCC1* protein is an important component of the BER pathway, which fixes base damage and DNA single‐strand breaks caused by ionizing radiation, alkylating agents, and oxidative damage 20, 21. Although its functional effect has not been well known, rs25487 G>A (R399Q), occurs in the poly (ADP‐ribose) polymerase binding domain of *XRCC1* gene, may affect complex assembly, and reduce DNA repair efficiency 22. In our study, we found that the *XRCC1* rs25487 A allele was associated with a bad survival outcome for advanced NSCLC patients. This result was consistent with the latest meta‐analysis in 2012, which used 22 articles, that suggested *XRCC1* rs25487 GA and AA genotypes could influence overall survival of lung cancer patients [GA vs. GG: HR (95% CI) = 1.23 (1.06–1.44); AA vs. GG: HR (95% CI) = 2.03 (1.20–3.45)] 23. Moreover, one study accomplished in Shenyang, China found the adjusted HRs for *XRCC1* rs25487 GA and AA genotype were 1.28 and 2.68 in 257 nonsmoking female lung adenocarcinoma patients, respectively 24. Two additional studies also reported *XRCC1* rs25487 A allele was associated with shorter MSTs and higher death risk 25, 26.Our study provided the consistent results supporting the reliability of results from the above studies.

The unfavorable effect of *XRCC1* rs25487 A allele was mainly seen among male patients and patients who underwent chemotherapy but without surgery and radiotherapy. One study reported that *XRCC1* rs25487 A allele was associated with poor prognosis in stage II‐IIIA and among older individuals 27. However, three studies carried out in 161 advanced NSCLC patients 5 and 82 advanced NSCLC patients 28 who underwent platinum‐based chemotherapy, as well as in 74 advanced NSCLC patients treated with platinum‐based chemotherapy and additionally received concomitant or sequential radiotherapy 29, respectively, failed to identify significant associations between *XRCC1* rs25487 and survival outcomes. These inconsistent results may be due to their smaller sample sizes and the differences in specific stage, pathology, and therapy among patients in different studies.

In our study, no significant associations were found for *RRM1* variants (rs11030918 and rs12806698) and survival outcomes of advanced NSCLC patients. This result was consistent with two studies in Korea of 158 never‐smokers with NSCLC 25 and 298 advanced NSCLC patients 30, as well as one study in China of 340 NSCLC patients 31, respectively. The latest meta‐analysis in 2012 using 10 cohort studies with a total of 1252 NSCLC patients assessed that neither *ERCC1* rs3212986 nor rs11615 variant had any influence on survival outcomes of platinum‐based treatment among advanced NSCLC patients 32. Another meta‐analysis in 2011 including 17 studies also found that neither *ERCC1* (rs3212986 and rs11615) nor *ERCC2* (rs13181) was significantly associated with response and progression‐free survival in NSCLC patients 33. Our study provided the similar negative associations between above variants and survival of advanced NSCLC patients. No significant correlations with survival outcomes were found in two studies for *XPA* rs1800975 34, 35 and nine studies for *XPG* rs17655 26, 27, 29, 31, 34, 35, 36, 37, 38, respectively. Our study provided the similar results. However, two studies indicated *XPA* rs1800975 GA/AA was significantly associated with poor NSCLC survival 29, 39. Additionally, no published investigations had provided clues among *XPB* (rs2276583), *XPF* (rs1799797), *CSB* (rs3793784), *DDB2* (rs3781619 and rs2029298), *FEN1* (rs174538 and rs4246215), *APEX1* (rs1130409) and the survival outcomes in NSCLC patients. The results of the above investigations suggested that there were inconsistent observations between different studies, and the reasons may be explained by the diversity of genetic background between Caucasians and Asians, different specific stage, pathology, and therapy and sample sizes.

There were several strengths in our study. Firstly, all lung patients were staff members of Wuhan Iron and Steel (Group) Corporation, who had a similar economic status, a better medical compliance, and high follow‐up rate (98%). Secondly, this study included 13 SNPs in nine NER genes and four SNPs in three BER genes for analysis, and all of them are important components in DNA repair pathways. However, some limitations of this study should not be neglected. Firstly, we used a moderate sample sized advanced NSCLC patients in the survival analysis, and additional studies with larger population were needed for further validation. In addition, because of lacking functional assays, the underlying biologic mechanisms for the observed positive SNPs are still unclear and need further investigation.

In conclusion, our study provided preliminary evidence that the *ERCC2* rs50872 T allele was associated with a favorable survival while the *XRCC1* rs25487 A allele was associated with a worse survival outcome for advanced NSCLC patients. Furthermore, advanced NSCLC patients carrying the *ERCC2* rs50872 C in combination with *XRCC1* rs25487 A allele rendered the shortest MST and highest death risk for advanced NSCLC patients. Additional studies carried out in lung cancer patients with specific stage, pathology, and therapy, as well as functional biological studies need to be validated for potential associations.

## Conflict of Interest

The authors declare that they have no conflict of interest.

## Supporting information


**Table S1**. Primers and probes used for TaqMan allelic discrimination.

## References

[1] Jemal, A. , F. Bray , M. M. Center , J. Ferlay , E. Ward , and D. Forman . 2011 Global cancer statistics. CA Cancer J. Clin. 61:69–90. 21296855 10.3322/caac.20107

[2] Herbst, R. S. , J. V. Heymach , and S. M. Lippman . 2008 Lung cancer. N. Engl. J. Med. 359:1367–1380. 18815398 10.1056/NEJMra0802714 PMC10662965

[3] Sekine, I. , N. Yamamoto , K. Nishio , and N. Saijo . 2008 Emerging ethnic differences in lung cancer therapy. Br. J. Cancer 99:1757–1762. 18985035 10.1038/sj.bjc.6604721 PMC2600690

[4] Wei, Q. , M. L. Frazier , and B. Levin . 2000 DNA repair: a double‐edged sword. J. Natl Cancer Inst. 92:440–441. 10716953 10.1093/jnci/92.6.440

[5] Du, Y. , T. Su , L. Zhao , X. Tan , W. Chang , H. Zhang , et al. 2014 Associations of polymorphisms in DNA repair genes and MDR1 gene with chemotherapy response and survival of non‐small cell lung cancer. PLoS ONE 9:e99843. 24933103 10.1371/journal.pone.0099843 PMC4059653

[6] Rosell, R. , R. V. Lord , M. Taron , and N. Reguart . 2002 DNA repair and cisplatin resistance in non‐small‐cell lung cancer. Lung Cancer 38:217–227. 12445742 10.1016/s0169-5002(02)00224-6

[7] Zhang, X. , X. Miao , G. Liang , B. Hao , Y. Wang , W. Tan , et al. 2005 Polymorphisms in DNA base excision repair genes ADPRT and XRCC1 and risk of lung cancer. Cancer Res. 65:722–726. 15705867

[8] Zienolddiny, S. , D. Campa , H. Lind , D. Ryberg , V. Skaug , L. Stangeland , et al. 2006 Polymorphisms of DNA repair genes and risk of non‐small cell lung cancer. Carcinogenesis 27:560–567. 16195237 10.1093/carcin/bgi232

[9] Olaussen, K. A. , A. Dunant , P. Fouret , E. Brambilla , F. Andre , V. Haddad , et al. 2006 DNA repair by ERCC1 in non‐small‐cell lung cancer and cisplatin‐based adjuvant chemotherapy. N. Engl. J. Med. 355:983–991. 16957145 10.1056/NEJMoa060570

[10] Tanaka, Y. , Y. Maniwa , V. P. Bermudez , T. Doi , W. Nishio , C. Ohbayashi , et al. 2010 Nonsynonymous single nucleotide polymorphisms in DNA damage repair pathways and lung cancer risk. Cancer 116:896–902. 20052722 10.1002/cncr.24850

[11] Xu, P. , L. Liu , J. Wang , K. Zhang , X. Hong , Q. Deng , et al. 2013 Genetic variation in BCL2 3′‐UTR was associated with lung cancer risk and prognosis in male Chinese population. PLoS ONE 8:e72197. 23977251 10.1371/journal.pone.0072197 PMC3745400

[12] Kiyohara, C. , K. Takayama , and Y. Nakanishi . 2006 Association of genetic polymorphisms in the base excision repair pathway with lung cancer risk: a meta‐analysis. Lung Cancer 54:267–283. 16982113 10.1016/j.lungcan.2006.08.009

[13] Kim, S. H. , G. W. Lee , M. J. Lee , Y. J. Cho , Y. Y. Jeong , H. C. Kim , et al. 2012 Clinical significance of ERCC2 haplotype‐tagging single nucleotide polymorphisms in patients with unresectable non‐small cell lung cancer treated with first‐line platinum‐based chemotherapy. Lung Cancer 77:578–584. 22608006 10.1016/j.lungcan.2012.04.016

[14] Zhou, W. , G. Liu , D. P. Miller , S. W. Thurston , L. L. Xu , J. C. Wain , et al. 2003 Polymorphisms in the DNA repair genes XRCC1 and ERCC2, smoking, and lung cancer risk. Cancer Epidemiol. Biomarkers Prev. 12:359–365. 12692111

[15] Qian, B. , H. Zhang , L. Zhang , X. Zhou , H. Yu , and K. Chen . 2011 Association of genetic polymorphisms in DNA repair pathway genes with non‐small cell lung cancer risk. Lung Cancer 73:138–146. 21195504 10.1016/j.lungcan.2010.11.018

[16] Feng, Z. , Y. Ni , W. Dong , H. Shen , and J. Du . 2012 Association of ERCC2/XPD polymorphisms and interaction with tobacco smoking in lung cancer susceptibility: a systemic review and meta‐analysis. Mol. Biol. Rep. 39:57–69. 21614524 10.1007/s11033-011-0710-9

[17] Xue, H. , Y. Lu , B. Lin , J. Chen , F. Tang , and G. Huang . 2012 The effect of XPD/ERCC2 polymorphisms on gastric cancer risk among different ethnicities: a systematic review and meta‐analysis. PLoS ONE 7:e43431. 23028453 10.1371/journal.pone.0043431 PMC3441548

[18] Zhang, E. , Z. Cui , Z. Xu , W. Duan , S. Huang , X. Tan , et al. 2013 Association between polymorphisms in ERCC2 gene and oral cancer risk: evidence from a meta‐analysis. BMC Cancer 13:594. 24330540 10.1186/1471-2407-13-594 PMC3878799

[19] Zhu, M. L. , J. He , M. Wang , M. H. Sun , L. Jin , X. Wang , et al. 2014 Potentially functional polymorphisms in the ERCC2 gene and risk of esophageal squamous cell carcinoma in Chinese populations. Sci. Rep. 4:6281. 25209371 10.1038/srep06281 PMC4160711

[20] Masson, M. , C. Niedergang , V. Schreiber , S. Muller , J. Menissier‐de Murcia , and G. de Murcia . 1998 XRCC1 is specifically associated with poly(ADP‐ribose) polymerase and negatively regulates its activity following DNA damage. Mol. Cell. Biol. 18:3563–3571. 9584196 10.1128/mcb.18.6.3563 PMC108937

[21] Vidal, A. E. , S. Boiteux , I. D. Hickson , and J. P. Radicella . 2001 XRCC1 coordinates the initial and late stages of DNA abasic site repair through protein‐protein interactions. EMBO J. 20:6530–6539. 11707423 10.1093/emboj/20.22.6530 PMC125722

[22] Park, J. Y. , S. Y. Lee , H. S. Jeon , N. C. Bae , S. C. Chae , S. Joo , et al. 2002 Polymorphism of the DNA repair gene XRCC1 and risk of primary lung cancer. Cancer Epidemiol. Biomarkers Prev. 11:23–27. 11815397

[23] Cui, Z. , Z. Yin , X. Li , W. Wu , P. Guan , and B. Zhou . 2012 Association between polymorphisms in XRCC1 gene and clinical outcomes of patients with lung cancer: a meta‐analysis. BMC Cancer 12:71. 22339849 10.1186/1471-2407-12-71 PMC3305620

[24] Yin, Z. , B. Zhou , Q. He , M. Li , P. Guan , X. Li , et al. 2009 Association between polymorphisms in DNA repair genes and survival of non‐smoking female patients with lung adenocarcinoma. BMC Cancer 9:439. 20003463 10.1186/1471-2407-9-439 PMC2803496

[25] Han, J. Y. , K. A. Yoon , J. H. Park , Y. J. Lee , G. K. Lee , J. H. Han , et al. 2011 DNA repair gene polymorphisms and benefit from gefitinib in never‐smokers with lung adenocarcinoma. Cancer 117:3201–3208. 21264830 10.1002/cncr.25863

[26] Lee, S. Y. , H. G. Kang , S. S. Yoo , Y. R. Kang , Y. Y. Choi , W. K. Lee , et al. 2013 Polymorphisms in DNA repair and apoptosis‐related genes and clinical outcomes of patients with non‐small cell lung cancer treated with first‐line paclitaxel‐cisplatin chemotherapy. Lung Cancer 82:330–339. 23973201 10.1016/j.lungcan.2013.07.024

[27] Butkiewicz, D. , M. Rusin , B. Sikora , A. Lach , and M. Chorazy . 2011 An association between DNA repair gene polymorphisms and survival in patients with resected non‐small cell lung cancer. Mol. Biol. Rep. 38:5231–5241. 21188533 10.1007/s11033-010-0674-1

[28] Sun, X. , F. Li , N. Sun , Q. Shukui , C. Baoan , F. Jifeng , et al. 2009 Polymorphisms in XRCC1 and XPG and response to platinum‐based chemotherapy in advanced non‐small cell lung cancer patients. Lung Cancer 65:230–236. 19157633 10.1016/j.lungcan.2008.11.014

[29] Sullivan, I. , J. Salazar , M. Majem , C. Pallares , E. Del Rio , D. Paez , et al. 2014 Pharmacogenetics of the DNA repair pathways in advanced non‐small cell lung cancer patients treated with platinum‐based chemotherapy. Cancer Lett. 353:160–166. 25069034 10.1016/j.canlet.2014.07.023

[30] Ryu, J. S. , E. S. Shin , H. S. Nam , H. G. Yi , J. H. Cho , C. S. Kim , et al. 2011 Differential effect of polymorphisms of CMPK1 and RRM1 on survival in advanced non‐small cell lung cancer patients treated with gemcitabine or taxane/cisplatinum. J. Thorac. Oncol. 6:1320–1329. 21642870 10.1097/JTO.0b013e3182208e26

[31] Ren, S. , S. Zhou , F. Wu , L. Zhang , X. Li , J. Zhang , et al. 2012 Association between polymorphisms of DNA repair genes and survival of advanced NSCLC patients treated with platinum‐based chemotherapy. Lung Cancer 75:102–109. 21676483 10.1016/j.lungcan.2011.05.023

[32] Yu, D. , J. Shi , T. Sun , X. Du , L. Liu , X. Zhang , et al. 2012 Pharmacogenetic role of ERCC1 genetic variants in treatment response of platinum‐based chemotherapy among advanced non‐small cell lung cancer patients. Tumour Biol. 33:877–884. 22249976 10.1007/s13277-011-0314-y

[33] Yin, M. , J. Yan , A. Voutsina , C. Tibaldi , D. C. Christiani , R. S. Heist , et al. 2011 No evidence of an association of ERCC1 and ERCC2 polymorphisms with clinical outcomes of platinum‐based chemotherapies in non‐small cell lung cancer: a meta‐analysis. Lung Cancer 72:370–377. 21075476 10.1016/j.lungcan.2010.10.011 PMC3050122

[34] Kim, M. , H. G. Kang , S. Y. Lee , H. C. Lee , E. B. Lee , Y. Y. Choi , et al. 2010 Comprehensive analysis of DNA repair gene polymorphisms and survival in patients with early stage non‐small‐cell lung cancer. Cancer Sci. 101:2436–2442. 20731661 10.1111/j.1349-7006.2010.01699.x PMC11159840

[35] Campayo, M. , N. Vinolas , A. Navarro , E. Carcereny , F. Casas , B. Gel , et al. 2011 Single nucleotide polymorphisms in tobacco metabolism and DNA repair genes and prognosis in resected non‐small‐cell lung cancer. J. Surg. Res. 167:e5–e12. 21324488 10.1016/j.jss.2011.01.007

[36] Zhang, T. , J. Sun , M. Lv , L. Zhang , X. Wang , J. C. Ren , et al. 2013 XPG is predictive gene of clinical outcome in advanced non‐small‐cell lung cancer with platinum drug therapy. Asian Pac. J. Cancer Prev. 14:701–705. 23621222 10.7314/apjcp.2013.14.2.701

[37] Mathiaux, J. , V. Le Morvan , M. Pulido , J. Jougon , H. Begueret , and J. Robert . 2011 Role of DNA repair gene polymorphisms in the efficiency of platinum‐based adjuvant chemotherapy for non‐small cell lung cancer. Mol. Diagn. Ther. 15:159–166. 21766907 10.1007/BF03256406

[38] Shiraishi, K. , T. Kohno , C. Tanai , Y. Goto , A. Kuchiba , S. Yamamoto , et al. 2010 Association of DNA repair gene polymorphisms with response to platinum‐based doublet chemotherapy in patients with non‐small‐cell lung cancer. J. Clin. Oncol. 28:4945–4952. 20940192 10.1200/JCO.2010.30.5334

[39] Butkiewicz, D. , A. Drosik , R. Suwinski , M. Krzesniak , M. Rusin , A. Kosarewicz , et al. 2012 Influence of DNA repair gene polymorphisms on prognosis in inoperable non‐small cell lung cancer patients treated with radiotherapy and platinum‐based chemotherapy. Int. J. Cancer 131:E1100–E1108. 22511383 10.1002/ijc.27596

