# Supplementary material for: The genetic variations in DNA repair genes ERCC2 and XRCC1 were associated with the overall survival of advanced non‐small‐cell lung cancer patients
Source: Cancer Med. 2016 Jul 27;5(9):2332–42. doi: 10.1002/cam4.822 (PMC5055187; doi:10.1002/cam4.822)
Supplement: Supplementary file 1 — Table S1. Primers and probes used for TaqMan allelic discrimination. [file CAM4-5-2332-s001.docx]

| **Supplementary Table 1. Primers and probes used for TaqMan allelic discrimination.** | | | |
| --- | --- | --- | --- |
| **Genes** | **dbSNP** |  | **Sequence（5′→3′）** |
| *RRM1* |  |  |  |
|  | rs11030918 | Primer_F | TCCATCCTACCTCCACAAGG |
|  |  | Primer_R | CGATGGCGTTTGGATTTTAT |
|  |  | Probe (FAM) | AGAGAATTTTAAGCAGG (MGB) |
|  |  | Probe (HEX) | AGAGGATTTTAAGCAGG (MGB) |
|  | rs12806698 | Primer_F | CTTGCCCAGACTCAACAT |
|  |  | Primer_R | CCAGACAGCACTTTCTTCAG |
|  |  | Probe (FAM) | TGTGAAGCCTACCCCG (MGB) |
|  |  | Probe (HEX) | TCTGTGAAGACTACCCC (MGB) |
| *ERCC1* | |  |  |
|  | rs11615 | Primer_F | AGGGGCAATCCCGTACTGA |
|  |  | Primer_R | CGGGAATTACGTCGCCAA |
|  |  | Probe (FAM) | CGTGCGCAACGTGCCCTG |
|  |  | Probe (HEX) | TCGTGCGCAATGTGCCCTG |
|  | rs3212986 | Primer_F | GGCACCTTCAGCTTTCTTTAGTTC |
|  |  | Primer_R | ACAGGCCGGGACAAGAAG |
|  |  | Probe (FAM) | TGCTGCTTCTTCC |
|  |  | Probe (VIC) | TGCTGCTGCTTCC |
| *ERCC2* | |  |  |
|  | rs13181 | Primer_F | AGGAGTCACCAGGAACCGTTTAT |
|  |  | Primer_R | AGGAGTCACCAGGAACCGTTTAT |
|  |  | Probe (FAM) | TCTGCTCTATCCTCTTCAGCGTCTCCTC |
|  |  | Probe (HEX) | CTGCTCTATCCTCTGCAGCGTCTCCT |
|  | rs50872 | Primer_F | CACTATTTCCCATCTGCCAACA |
|  | | Primer_R | AGGGCTTCCTGGAGGACAAG |
|  | | Probe (FAM) | CCTTAGGCTCTCAGC |
|  | | Probe (HEX) | CATCCTTAGGTTCTCAG |
| *XPB* | |  |  |
|  | rs2276583 | Primer_F | GAGCAGGGACCACAGTCAACTAC |
|  |  | Primer_R | CCAGGGAGCTACCAGAGAGATG |
|  | | Probe (FAM) | CACCCTCCCCGTTCCCCAG |
|  | | Probe (HEX) | CACCCTCCCCATTCCCCAGG |
| *XPF* | |  |  |
|  | rs1799797 | Primer_F | CACCAGCTGTCGCTCGTACTC |
|  | | Primer_R | TCGGCCCACGATCATCTC |
|  | | Probe (FAM) | AGCCGAACGCAGCCGAAGG |
|  | | Probe (HEX) | AGCCGAACGCTGCCGAAGG |
| *CSB* | |  |  |
|  | rs3793784 | Primer_F | AACGCTGGACTTAAGGCAATGT |
|  | | Primer_R | GCCAGGCTGTGACCTCTCTT |
|  | | Probe (FAM) | AGGACAGCTCTCCATCCTTCCCG |
|  | | Probe (HEX) | AGCAGGACAGCTCTGCATCCTTCC |
| *XRCC1* |  |  |  |
|  | rs25487 | Primer_F | GAGTGGGTGCTGGACTGTCA |
|  |  | Primer_R | CATTGCCCAGCACAGGATAAG |
|  |  | Probe (FAM) | CTGCCCTCCCGGAGGTAAGGC |
|  |  | Probe (HEX) | CTGCCCTCCCAGAGGTAAGGCC |
| *XPA* | rs1800975 | C___482935_1 |  |
| *XPG* | rs17655 | C___1891743_10 |  |
| *DDB2* | rs2029298 | C___1865812_10 |  |
|  | rs3781619 | C___30888105_10 |  |
| *FEN1* | rs174538 | C___2575553_10 |  |
| *APEX1* | rs1130409 | C___8921503_10 |  |
